# Genome Size Diversity and Its Impact on the Evolution of Land Plants

**DOI:** 10.3390/genes9020088

**Published:** 2018-02-14

**Authors:** Jaume Pellicer, Oriane Hidalgo, Steven Dodsworth, Ilia J. Leitch

**Affiliations:** Department of Comparative Plant and Fungal Biology, Royal Botanic Gardens, Kew TW9 3DS, UK; o.hidalgo@kew.org (O.H.); s.dodsworth@kew.org (S.D.); i.leitch@kew.org (I.J.L.)

**Keywords:** genome size, polyploidy, transposable elements, C-value, giant genome

## Abstract

Genome size is a biodiversity trait that shows staggering diversity across eukaryotes, varying over 64,000-fold. Of all major taxonomic groups, land plants stand out due to their staggering genome size diversity, ranging ca. 2400-fold. As our understanding of the implications and significance of this remarkable genome size diversity in land plants grows, it is becoming increasingly evident that this trait plays not only an important role in shaping the evolution of plant genomes, but also in influencing plant community assemblages at the ecosystem level. Recent advances and improvements in novel sequencing technologies, as well as analytical tools, make it possible to gain critical insights into the genomic and epigenetic mechanisms underpinning genome size changes. In this review we provide an overview of our current understanding of genome size diversity across the different land plant groups, its implications on the biology of the genome and what future directions need to be addressed to fill key knowledge gaps.

## 1. Introduction

Genome size (hereafter GS) is widely used to refer to the amount of DNA contained in the cell nucleus. Also known as the C-value, it refers to the “holoploid genome size” (i.e., the DNA content of the unreplicated gametic chromosome complement = 1C-value), which is typically broadly constant within an organism [1,2]. Since the early studies of the 1950s and 1960s, this trait has underpinned numerous research programmes aiming to investigate the origin, extent and most importantly, the biological significance of GS diversity and its impact on the evolution of eukaryotes. Today, the relevance of GS as a biodiversity trait is clear, given that available data for over 15,000 species of eukaryotes shows an astonishing range varying over 64,000-fold [3,4,5]. Out of all these data, land plants stand out because they are the most widely studied group (with values for over 12,000 species), and because of their remarkable diversity, ranging ca. 2400-fold—the largest range for any comparable group of organisms (see Hidalgo et al. [6] for an outline of the technical issues and poor reliability of the giant genome sizes previously estimated for some amoebae and dinoflagellates). Nevertheless, there is still much to be uncovered. In the age of high-throughput sequencing technologies, C-values provide, for example, critical data for sequencing projects. More interesting, however, are the insights being gained from the analysis of huge amounts of sequence data that shed new light on the molecular composition, dynamics and evolution of plant genomes of all sizes [7]. In this review we provide an overview of our current understanding of genome size diversity across the different land plant groups, its implications on the biology of the genome and what future directions need to be addressed to fill key knowledge gaps. 

## 2. Genome Size Diversity across Land Plants: What Do We Know?

**Figure 1:**
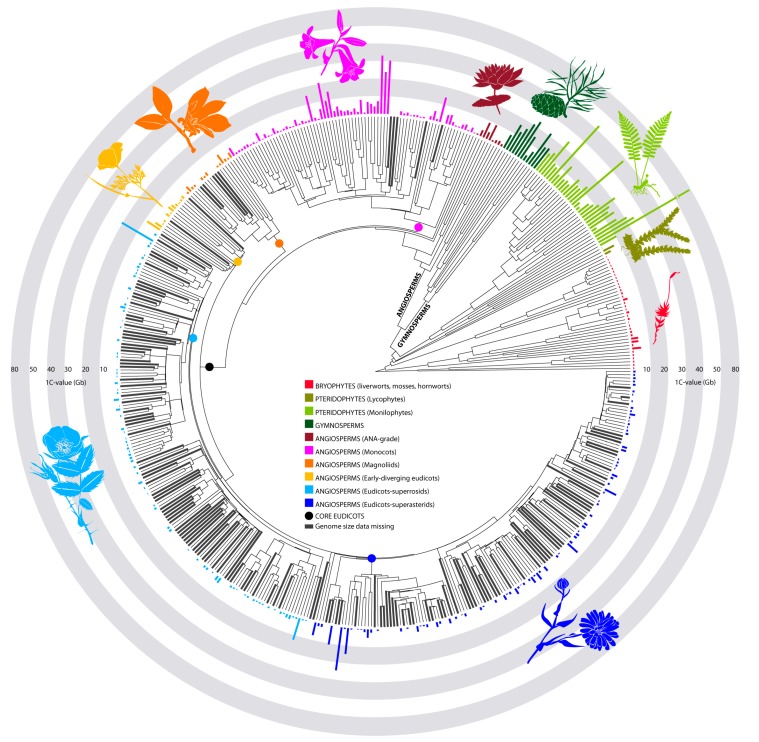
Phylogenetic tree illustrating the main taxonomic lineages across land plants summarized at the family level (extracted from Zanne et al. [14]). Average GS values are depicted for each family and branches leading to families without any GS data are shown in bold. (ANA-grade refers to species belonging to the three early-diverging angiosperm orders Amborellales, Nymphaeales and Austrobaileyales [12]).

According to the fossil record, plants first colonized terrestrial environments back in the middle Ordovician ca. 450–470 Mya [8,9], and have since diversified into four major land plant groups that persist to this day. These comprise: (i) bryophytes (including liverworts, mosses and hornworts); (ii) lycophytes, the earliest group to develop a vascular system ca. 400 Mya; (iii) the monilophytes, which include horsetails (*Equisetum*), whisk ferns (Ophioglossales and Psilotales), and true ferns; and (iv) the two extant seed plant lineages, gymnosperms and angiosperms (i.e., flowering plants), the latter currently representing the most ecologically successful and diverse land plant group on earth (Figure 1). While there has been a significant increase in GS data in recent years [3,10], our understanding of GS diversity across these different lineages is still somewhat biased (Table 1), and caution needs to be applied when undertaking comparative analyses of the dynamics of GS evolution between different land plant groups and over long evolutionary time scales. Certainly, taxonomic gaps are particularly notable in angiosperms, where nuclear DNA contents are only available for ca. 2100 genera out of the ca. 14,000 genera currently recognized, and with estimates for 10,768 species, data are available for just ca. 3% of the ca. 352,000 species recognized [11]. At the family level, this translates into estimates for about 267 families out of the 416 listed in the latest revision of the Angiosperm Phylogeny Group (APG IV) [12], i.e., only 64% of currently accepted families (Figure 1). Nonetheless, research that expands our understanding of GS diversity and evolution across land plants has increased significantly in recent years, uncovering remarkable differences in the genomic profiles of the different land plant lineages (Figure 1) [3,13]. Certainly, the continued growth in available data is providing novel opportunities to conduct more robust comparative analyses and therefore move the field forward.

Despite the caveats outlined above, available data have already revealed a staggering diversity of GSs across land plants (ranging ca. 2440-fold and differing by ca. 148,000 Mb). This is in striking contrast to the remaining eukaryotic groups that have been studied so far, where differences in GS are frequently orders of magnitude lower [4,15]. At the lower end of the scale, the smallest land plant genomes so far reported are found in species belonging to two distinct lineages: (i) *Genlisea tuberosa* (Lentibulariaceae, 61 Mb/1C) [16], a carnivorous angiosperm endemic to Brazil; and (ii) *Selaginella selaginoides* (Selaginellaceae, 78 Mb/1C) [17], a spikemoss that belongs to the lycophytes. At the upper end, the largest genome reported so far belongs to the angiosperm *Paris japonica*, a monocot lily in the Melanthiaceae family. It has an astonishingly large genome made up of ca. 149,000 Mb/1C of DNA [18]. Until recently, other plant species with giant genomes greater than 100,000 Mb/1C (i.e., *Trillium*, *Fritillaria* (Liliales) and *Viscum* (Santalales)) [6] were only found among the angiosperms. However, the recent discovery of genomic gigantism in the whisk-fern *Tmesipteris obliqua* (Psilotales, a monilophyte) with ca. 147,000 Mb/1C [19], provides robust evidence that, although scarce, giant genomes have evolved independently more than once across the plant tree of life. Such giant genomes in plants are most frequently the result of a combination of polyploidy (i.e., whole genome duplications, WGD) and high levels of repetitive DNA amplification combined with low rates of DNA removal [18,19,20,21] (see below). There are however, potential exceptions such as *Viscum album* (100,600 Mb/1C) [22], where a likely polyploid origin for its giant genome remains to be confirmed. Regardless of the origin and taxonomic group, the synthesis of available GS data shows that giant genomes in eukaryotes tend to converge at the higher end, suggesting that a series of evolutionary forces are acting to prevent genomes from expanding much beyond 150,000 Mb, and this has led to the suggestion that an upper limit to GS may have been reached [6].

**Figure 2:**
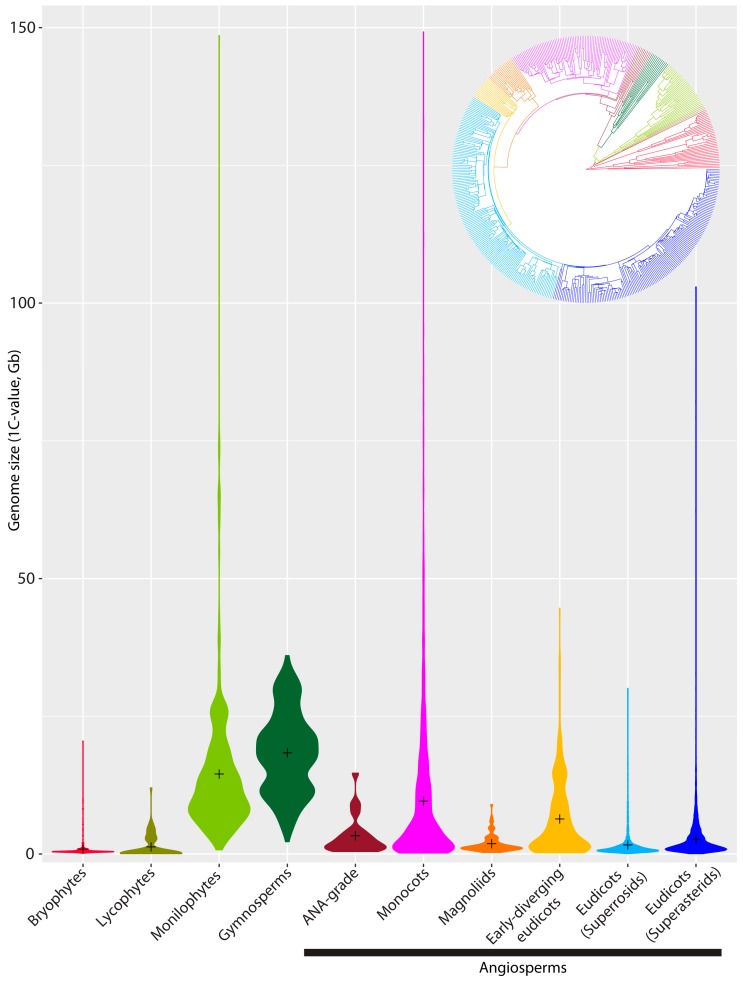
Violin plots illustrating the frequency and range of GS across the major land plant lineages. N.B. the taxonomic groups and colour schemes reflect the same lineage diversity as depicted in Figure 1. (ANA-grade refers to species belonging to the three early-diverging angiosperm orders Amborellales, Nymphaeales and Austrobaileyales [12]).

While large and giant genomes are clearly rare in all groups of land plants, the different propensities for genome expansion (via polyploidy and/or repeat amplification—see Section 3 below) versus genome contraction (e.g., via various recombination-based mechanisms such as illegitimate recombination—see Section 3 below) has resulted in contrasting distributions of GSs at the lower end of the scale (Figure 2). For example, the strongly skewed distribution in GS towards small and very small genomes in angiosperms (modal 1C-value = 587 Mb, mean 1C-value = 5020 Mb, Table 1) contrasts with monilophytes where the distribution is less skewed (modal and mean values are ca. 20× and ca. 3× higher, respectively), and the smallest record available (i.e., *Azolla microphylla* 1C = 748 Mb) is ca. 10× larger than the smallest record for an angiosperm. In addition, a large-scale phylogenetically-informed analysis of GS and chromosome number evolution in ferns [23] revealed that GS was significantly correlated with chromosome number across the vast majority of ferns analyzed (reflected in a general tendency for DNA content per chromosome to be conserved) despite a high frequency of polyploid events throughout their evolution. This contrasts with observations in angiosperms, where polyploidy is also frequent but is typically accompanied by subsequent genomic restructuring leading to a reduction in chromosome number and GS towards a more stable diploid-like genomic state [24,25].

Gymnosperms are another intriguing group in relation to GS diversity (Figure 2), contrasting to both angiosperms and monilophytes. They are currently the best represented land plant lineage in terms of available data (GS data for 41% of gymnosperm species, 95% of genera and all 12 families, [3]). These data show that gymnosperm genomes are, on average, larger than any other major land plant lineage, with a more gaussian-like distribution of values rather than being skewed towards the lower end of the scale (Figure 2, Table 1). Nevertheless, when it comes to how much GS diversity is contained within gymnosperms, this is probably one of the most conserved lineages, with C-values ranging only 16-fold (Table 1). The question then arises—why are gymnosperm genomes typically larger than those of most other land plants, and what evolutionary selective pressures might be favouring their larger sizes? There are several genomic characteristics that set gymnosperms apart from other land plant groups. These include: (i) the relatively narrow range of chromosome numbers and on, average larger sizes (i.e., 2n = 14–66, mean DNA amount/chromosome = 1.8 pg) compared with, for example, angiosperms (2n = 4–ca. 640, mean DNA amount/chromosome = 0.5 pg) and monilophytes (2n = 18–ca. 1440, mean DNA amount/chromosome = 0.2 pg); (ii) a remarkable constancy in chromosome numbers and karyotypes within genera and families [26] (e.g., all but three of the 156 chromosome counts reported for Pinaceae by Murray [26] are 2n = 24); and (iii) polyploidy is both rare (e.g., multiple cytotypes and/or allopolyploidy reported in just a few genera such as *Ephedra* [27,28], *Juniperus* [29], *Ginkgo* [30] and *Sequoia* [31]) and the maximum ploidal level reached (i.e., octoploidy in *Ephedra*) is considerably lower than in angiosperms (maximum ploidal level = 80× in *Sedum*) and monilophytes (maximum ploidal level = 96× in *Ophioglossum*); (iv) the lowest recombination rates so far reported in any eukaryotic lineage [32,33]. Recent insights gained from whole genome sequencing of various conifers [34,35,36] and *Ginkgo biloba* [37] have further highlighted the distinctive nature of the genomic landscape in these gymnosperms compared with angiosperms that have small to medium sized genomes. The data reveal that these large conifer and *Ginkgo* genomes, which contain a huge wealth of divergent and ancient repeats, including retrotransposons, have most likely arisen via a combination of ongoing repeat amplification over long periods of evolutionary time [34,35], combined with a lack of efficient and/or slower rates of repeat elimination via recombination-based processes [36,38]. Nevertheless, analysis of the recently published whole genome sequence of *Gnetum montanum* (Gnetales), which has one of the smallest GS of any gymnosperm so far reported (4.2 Gb/1C), cautions against generalizing the genomic insights gained from conifers and *Ginkgo* to all gymnosperms. Instead, probing the *Gnetum* genome shows that while it is also full of diverse, ancient repeats (especially retrotransposons), suggesting a similar profile of repeat accumulation as in conifers and *Ginkgo*, a higher frequency of repeat elimination via unequal recombination is apparent, which likely contributes to its considerably smaller GS [39].

## 3. Mechanisms Contributing to Genome Size Changes and its Impact on the Structure of the Genome

As introduced earlier, it is widely known that the main mechanisms contributing to genome expansion are polyploidy and the accumulation of repetitive DNA sequences that make up the bulk of the genome. Typically, these processes are, however, efficiently counterbalanced by machinery that prevents genomes from uncontrolled expansion, and which can lead to an overall reduction in the size of the genome. Amongst them, transposon-mediated unequal homologous recombination, illegitimate recombination and deletion-biased double strand break (DSB) repair pathways are frequently invoked as the likely causes of genome shrinkage, (reviewed in Schubert and Vu, [40]). Nevertheless, the relative contribution of these different mechanisms seems to vary between species. For example, a genome wide analysis of *Arabidopsis thaliana* suggested that illegitimate recombination was the main driving force contributing to its small genome size [41], whereas an analysis of the carnivorous plant *Genlisea nigrocaulis* by Vu et al. pointed to the importance of deletion biased DSB repair in underpinning genome shrinkage in this species [42]. Indeed, Vu et al. went on to suggest that such a mechanism may well play a more significant role in plant genome size evolution than hitherto recognized, given the ubiquitous occurrence of DSB repair, the evolutionary impact of misrepair events on plant meristems and their potential heritability.

Polyploidy has a direct impact on GS and—even in the absence of hybridization—it also enhances genetic diversity and genomic dynamism; these can provide new opportunities for gene neofunctionalization and subfunctionalization [43,44]. Together, these processes have generally been considered as facilitators of adaptation to environmental change and subsequent speciation [45]. Furthermore, recent research into the genomic landscape of minute genomes suggests that recent polyploidy events may also play a counteracting role to genome downsizing, hence preventing the loss of pivotal genes in extreme cases [42].

In the absence of polyploidy, genomes can expand through the accumulation of different types of repetitive elements, including DNA transposons, satellite DNAs and retroelements. In plants, the Ty3/Gypsy and Ty1/Copia retrotransposon superfamilies are usually the most abundant repetitive elements, though the dominance of one type or another is variable between taxa. For example, in the Fabeae tribe of Leguminosae, genome dynamics are dominated by a single lineage of Ty3/Gypsy elements—the Ogre chromovirus-type elements—that account for 57% of the variation in GS in this clade [46]. In many groups, the Ty3/Gypsy elements appear to dominate the genomic landscape, by being both the most abundant elements but also those that are particularly dynamic in terms of copy number, e.g., in *Nicotiana* [47,48] and *Solanum* [49]; Orobanchaceae [50]; *Asclepias* [51]; *Musa* [52]. What makes the chromovirus-type of Ty/Gypsy elements particularly prone to accumulate in plant genomes is hitherto unclear, but thought to be related to the chromodomain, which can enable targeting of these elements to regions of heterochromatin where they are less likely to be removed by recombination-based processes (e.g., centromeric regions).

Genome dynamics of repetitive sequences can make a large contribution to genome evolution at a variety of levels. This includes the structural organization and broad composition of the genome, epigenetic effects, and also the more fine-tuned regulation of gene expression and gene space [53]. At the broader architectural scale, the number and type of different repeats and their epigenetic status have been shown to impact the overall dynamics and balance between genome expansion versus contraction. For example, in the giant genomes of the angiosperm *Fritillaria* (Liliaceae; 1C-values range from ca. 30 to 100 Gb), many diverse repeats are present, each in relatively low abundance, with no particular element dominating the genome [20]. Additionally, each repeat family is fairly heterogeneous, which suggests they have been steadily accumulating slightly different copies over time rather than arising from recent bursts of activity. Thus the expansion of these giant genomes is potentially due more to a failure to remove DNA, than from the massively increased activity of one or more repetitive element families [20]. As mentioned in the previous section, gymnosperms generally have larger genomes in which the same pattern (i.e., a diverse sets of repeats) as described in *Fritillaria* has been reported. However, the mechanisms underlying this genomic structure are still unclear; although there are hints that there are differences between the epigenetic pathways of gymnosperms and angiosperms, and indeed between genomes of different sizes [54,55]. 

There are contrasting hypotheses to explain how genomes might grow to gigantic scales, but they are likely to involve, at least in part, differences in the efficiency of the epigenetic machinery. It is suggested that above a certain threshold of genome size, large tracts of heterochromatin are formed from stretches of repeats that become ‘locked down’ into highly condensed chromatin through the activity of the epigenetic machinery. Such a mechanism will not only reduce the potentially negative impact of transposon activity, but will also limit the accessibility of the recombination machinery, and hence the potential of recombination-based processes to eliminate DNA [56]. In support of this, recent studies have shown that (i) the lowest rates of unequal recombination between the long terminal repeats of long terminal repeat (LTR) retrotransposons were found in the largest genomes analyzed (maize and conifers) [38], while (ii) a preliminary analysis of the epigenetic pathways (e.g., RNA directed DNA methylation, RdDM) known to be involved in silencing repeat activity in species with smaller genomes were shown to be active but potentially operating in subtly different ways in the giant genomes of *Fritillaria* compared with smaller genomed species [57]. Greater insights into the role of epigenetic pathways—including the RdDM—to suppress activity of transposable elements (TE) as well as other counterbalancing mechanisms is an active area of research [58], and hopefully this will provide novel evidence as to whether or not they are universally applicable to species across the diversity of genome sizes and land plant lineages, as this is currently unknown. 

## 4. The Impact of Genome Size in Plant Diversity: From Species to Ecology

It has long been recognized that a plant’s GS has implications for many aspects of its biology through its impact on size and rate-related traits at the nuclear, cellular and whole plant levels, and ultimately influencing how and where plants grow and their ability to persist over evolutionary time scales [59,60,61]. Recent advances in methodological and statistical approaches are now making it possible to revisit some of the earlier findings, mostly regarding the constraints posed by GS, in an experimental setup. In particular, (i) the development of modern statistical modelling approaches incorporating the use of phylogenetic frameworks (e.g., [62]), (ii) the availability of extended datasets, higher data quality standards, and robust experimental design for hypothesis testing at different levels; from within-species (e.g., *Zea mays*) [63] up to large-scale ecosystem patterns such as e.g., mangroves [64] and grassland community dynamics [62], and (iii) a better understanding of the molecular mechanisms responsible for GS variation (as outlined in Section 3), are all contributing to a more robust understanding of the complex interplay between genomic, epigenetic and ecological factors that influence how GS impacts the evolutionary trajectory of a plant and its ability to respond to environmental change. 

From a genomic perspective, there have been several studies that have taken advantage of increasingly large GS datasets and increased amounts of molecular data to explore whether GS itself is playing a role in influencing the rate of plant molecular evolution and hence speciation. However, the findings are somewhat contradictory. Bromham et al. [65] analyzed data from 139 angiosperm families and reported that GS itself was negatively correlated with rates of molecular evolution, hence suggesting that small GSs could have a broader evolvability spectrum. In contrast, Puttick et al. [66] analyzed a dataset comprising 3351 species from across all land plants and concluded that the relationship between GS and speciation lies more closely with the rates of GS change—and not with the absolute GS. This would then place an emphasis on the mechanisms of GS change (e.g., polyploidization, post-polyploidization diploidization and transposable element activity) as key players promoting evolutionary novelties and reproductive barriers, and hence the evolution of new species. Further studies are clearly needed, especially given that many genomic processes (e.g., frequency of polyploidy, rates of recombination, efficiency in eliminating non-coding repetitive DNA sequences) underpinning the evolution of different land plant groups are increasingly being shown to be distinctive [17,33,67], thus the impact of GS on speciation may be distinctive for each land plant group [13].

Certainly, the question as to whether GS is under selective constraint has been a subject of debate for quite some time. Different evolutionary models have been invoked regarding GS as a varying trait, which include neutral or effectively neutral variation models [68], maladaptive [69] and adaptive models arising from the apparent selective significance of correlations between GS and e.g., phenotypic traits [70]. In addition, the importance of genetic drift in contributing to GS evolution is also an important question under debate. Under the neutral model, extra DNA would be considered as maladaptive, but carried over passively between generations by random drift. By contrast, the proportional model of GS evolution considers that the rate of GS change is proportional to the size of the genome, and so faster rates of change would take place in larger genomes. If that assumption holds true, then one would predict that it would be more difficult for small genomes to become and remain larger, but easier for those at the upper end of the spectrum to become smaller [71]. While it is easy to understand the selective advantages of having a small GS, this is not the case for the large or very large genomes. Bearing in mind the biological costs of having a large genome, is there any chance that they might be under “directional” selection? Under a purely genetic drift scenario, the answer is clearly not. Based on the mechanisms outlined above, whereby giant genomes are more likely to arise due to a lack of DNA removal [20], one might assume that GS is likely to be driven by selection in the lower GS range, whereas increases could result from the inability of purifying selection to counteract the accumulation of DNA. Thus, instead of reaching a “GS optimum”, the genome would evolve towards its “highest tolerable maximum GS” [72], and this implies an increasingly restricted range of life strategies and ecological options compatible with a larger GS. There are repeated observations where the phenotypic and ecological spectrums are much broader in plants with small genomes [73], with larger genomes persisting only under conditions where selective pressures are more relaxed, and the life strategies are compatible with both the abiotic (e.g., water and nutrient supply) and biotic (e.g., cell division rates) limitations and demands imposed by possessing large genomes [19,74,75].

Recent studies have also shown that genomic landscapes, and therefore plant community assemblies, are influenced by nutrient availability [62,76]. These observations likely arise from the fact that nucleic acids are amongst the most nitrogen (N) and phosphorous (P) rich molecules of the cell, so under limiting nutrient conditions (particularly N and P), one might predict that species with large genomes, which are more demanding and costly to build and maintain than species with small genomes, would be less competitive. The studies by Guignard et al. [62] and Šmarda et al. [76] have both provided robust evidence in support of this. They showed that species with larger GS (particularly polyploid species) were only able to compete successfully and dominate plant communities when high levels of both N and P were present. The results suggest that there may be negative selection against species with large GS in nutrient-limited conditions. This has relevant eco-evolutionary implications, especially if we take into account the impact that human activities can have on ecosystems (e.g., via N deposition). Certainly, a better understanding of the interactions between nutrient dynamics, GS and polyploidy will be necessary to anticipate and make global predications of how species with different GS will respond and survive in the long term.

## 5. Concluding Remarks and Future Directions

As our understanding of the implications and significance of the amazing GS diversity in land plants grows, it is becoming self-evident that this trait plays not only an important role in shaping the evolution of plant genomes, but also in influencing plant community assemblages at the ecosystem level. It is, however, essential to continue building a comprehensive dataset of GS across all major taxonomic groups, particularly focusing on those lineages in which we currently lack or have very limited data. Whilst the available data show that most land plants possess small genomes, future studies may reveal the existence of additional giant genomes. Recent advances and improvements in novel sequencing technologies, as well as analytical tools, make it possible to gain critical insights into the genomic and epigenetic mechanisms governing GS changes. By combining efforts to (i) increase sampling and (ii) develop new modelling and analytical approaches, we will be ideally placed to tackle, trace and better understand the genomic signature and ecological significance of the processes responsible for generating the huge GS diversity of land plants. 

## Figures and Tables

**Table 1:** Minimum (Min.), maximum (Max.), and mean, median and modal 1C-values for each land plant group represented in the Plant DNA C-values database (Release 7.0), together with range in GS (genome size) and percentage representation of species in each group.

	Min. (Mb)	Max. (Mb)	Mean (Mb)	Mode (Mb)	Median (Mb)	Range in Absolute GS (Mb)	Range (Max./Min.)	Approx. no. of Species Recognised	No. of Species in the Plant DNA C-Values Database	Approx. % Species Representation in the Plant DNA C-Values Database
**Bryophytes**										
Hornworts	156	714	244	176	205	558	4-fold	250	23	9.2
Liverworts	206	20,010	1844	740	751	19,804	97-fold	5,000	102	2.0
Mosses	170	2004	504	442	433	1834	12-fold	12,000	184	1.7
**Pteridophytes**										
Lycophytes	78	11,704	1165	117	127	11,626	150-fold	900	57	6.3
Monilophytes	748	147,297	14,320	12,073	11,110	146,549	196-fold	11,000	246	2.2
**Spermatophytes**										
Gymnosperms	2201	35,208	17,947	21,614	21,614	33,007	16-fold	1026	421	41.0
Angiosperms	61	148,852	5020	587	1663	148,791	2440-fold	352,000	10,768	3.1
